# Supplementary material for: Estimating the effects of crime maps on house prices using an (Un)natural experiment: A study protocol
Source: PLoS One. 2022 Dec 1;17(12):e0278463. doi: 10.1371/journal.pone.0278463 (PMC9714891; doi:10.1371/journal.pone.0278463)
Supplement: S1 File — (DOCX) [file pone.0278463.s001.docx]

# S1. Example of analysis (RQ1)

For this analysis, we’ll use house price data for the year 2010 - 2013 and futher filter the sample according to the routine described in this protcol. For each house sold, we know the total number of potential snap points nearby (150m), the sold price (logged), and other characteristics. We use version one of the police.uk snap points list.

We report the means of key characteristics such as price sold and the coordinates of the property. There is little meaningful difference in these characteristics across the year (as we will later also prove).

year ppSnapsNearby price lnPrice oseast1m osnrth1m
 <chr> <dbl> <dbl> <dbl> <dbl> <dbl>
1 2009 3.81 130528. 11.7 443636. 397258.
2 2010 3.78 134210. 11.7 443647. 397321.
3 2011 3.72 126985. 11.7 443558. 397461.
4 2012 3.66 131079. 11.7 443548. 396802.
5 2013 3.64 131535. 11.7 443814. 397058.

## Estimation

**Estimator 1A: Non-parametric**

*Check whether the correlation (i.e. spearman’s rank) between* $Y$ *and* $M_{s}$ *is the same at* $T=0$ *and* $T=1$*. The null hypothesis is that the correlation remains the same. We can use bootstrapping or permutation tests to get standard errors.*

We can compute the rank correlation between house prices for these years:

2009 2010 2011 2012 2013
-0.2248691 -0.2317255 -0.1913800 -0.2101135 -0.2260731

There is a negative correlation between snaps and prices but this correlation reduces in 2011 – the year of police.uk’s launch. Under the null hypothesis, correlation across all year is the same. To simulate this, we can randomise the year that a house was sold and calculate the resulting correlation. We try this 1,000 times and get the lowest and highest 2.5% values of the correlation for each year:

`2.5%` `97.5%`
 <dbl> <dbl>
2009 -0.236 -0.198
2010 -0.235 -0.198
2011 -0.235 -0.198
2012 -0.234 -0.198
2013 -0.232 -0.200

For the year 2011, the rank correlation -0.191 falls above the highest 2.5% value under the null hypothesis (-0.198). Therefore, we can reject the null hypothesis that police.uk had no effect on house prices.

**Estimator 1B: Parametric estimation**

*Assuming that a linear functional form is close enough approximation, estimate the following model using OLS:*

$$E(Y)=\beta_{0}+\beta_{T}T+\beta_{M_{s}}M_{s}+\beta_{TM_{s}}T.M_{s}$$

*Under the null hypothesis that police.uk had no effect, we would expect* $\beta_{TM_{s}}$ *to be zero.*

Using the year 2010 and 2011 as $T=0$ and $T=1$ respectively; we can simply estimate the above regression equation. The resulting interaction term has a value of 0.009 ($p<0.05$). This shows again that we can reject the null hypothesis.

t test of coefficients:

 Estimate Std. Error t value Pr(>|t|)
(Intercept) 11.9073632 0.0107363 1109.0721 < 2.2e-16 ***
ppSnapsNearby -0.0540811 0.0024818 -21.7910 < 2.2e-16 ***
I(year == 2011)TRUE -0.0854014 0.0149388 -5.7167 1.103e-08 ***
ppSnapsNearby:I(year == 2011)TRUE 0.0085299 0.0034954 2.4404 0.01468 *

We can also add in LSOA fixed effect to the estimator. The resulting interaction term has a value of 0.006 ($p<0.05$). The OLS and fixed effect estimates are both within 1 standard error of each other.

t test of coefficients:

 Estimate Std. Error t value Pr(>|t|)
ppSnapsNearby -0.0334160 0.0032100 -10.4100 < 2.2e-16 ***
I(year == 2011)TRUE -0.0681298 0.0129909 -5.2444 1.586e-07 ***
ppSnapsNearby:I(year == 2011)TRUE 0.0063203 0.0029405 2.1494 0.03162 *

**Estimator 1C: Parametric time series**

*Assume that a linear functional form is close enough approximation. Then redefine* $T$ *to be an index number for year (e.g.*$T=2011$ *if the year is 2011). For every year, estimate the following model using OLS:*

$$E(Y_{T})=\beta_{0,T}+\beta_{M_{s},T}M_{s}$$

*The statistical association between* $Y$ *and* $M_{s}$ *(*$\beta_{M_{s},T}$*) may change over time. If that change was consistent (e.g. greater effect sizes due to inflation) then, for every year before 2011, we can fit the following model using OLS:*

$$E(\beta_{M_{s},T})=\delta_{0}+\delta_{T}T$$

*We can use the model to estimate* $\tilde{\beta}_{M_{s},2011}$*. This is what* $\beta_{M_{s},2011}$ *would have been if police.uk did not launch in 2011. Under the null hypothesis:*

$$\beta_{M_{s},2011}-\tilde{\beta}_{M_{s},2011}=0$$

For this estimator, we take data on houses sold since 1995 until 2019 (prior to COVID19). Run OLS for each year and plot the estimated parameter above. We use both price and logged price. The results are plotted below:


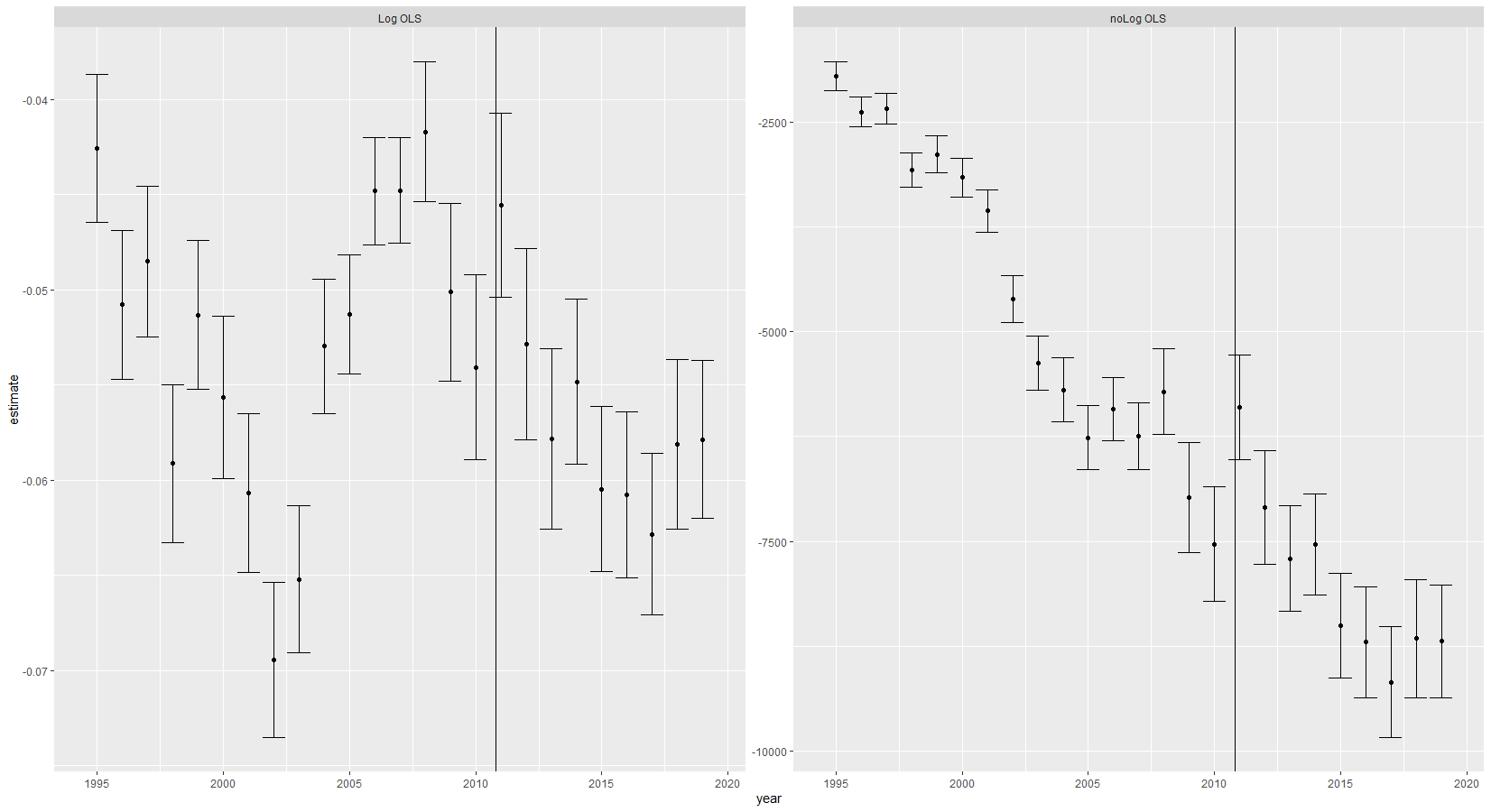
 **Figure S1.1: Time series of OLS estimates using price (logged, left) and price (untransformed, right)**

The error bars represent the 95% margin of error for each OLS estimate. We use OLS to predict the trend in these points between 1995 and 2010 (see below).

==========================================================
 Dependent variable:
 ----------------------------
 estimate
 (Log) (noLog)
----------------------------------------------------------
year 0.0002 -372.347***
 (0.0004) (24.618)

Constant -0.449 741,019.800***
 (0.881) (49,297.900)

----------------------------------------------------------
Observations 16 16
R2 0.014 0.942
Adjusted R2 -0.056 0.938
Residual Std. Error (df = 14) 0.008 453.936
F Statistic (df = 1; 14) 0.202 228.763***
==========================================================
Note: *p<0.1; **p<0.05; ***p<0.01

Between 1995-2010, there is no time trend using logged prices and a very predictable linear time trend using un-logged prices. The model fit for the untransformed house price model is very high ($R^{2}=0.94$). A very predictable time trend (or lack of one entirely) is a good sign.

Using the 1995-2010 OLS models, we predict a values of $\tilde{\beta}_{M_{s},2011}$ of 0.051 for logged prices and -7,800 for the un-logged prices. The real values of $\beta_{M_{s},2011}$ are -0.046 (se: 0.002) and -5,900 (se: 318). The difference between $\tilde{\beta}_{M_{s},2011}$ and $\beta_{M_{s},2011}$ (as well as the graph above) suggests that 2011 bucks the trend.

## Assumption testing

Take the coordinates, Eastings and Northings (nearest metre), of housing sold in South Yorkshire as potential confounders. Due to data limitations, we use the coordinates of the postcode centroid instead of the coordinates of the house itself. Let $T=0$ refer to the year 2010 and $T=1$ refer to the year 2011.

Assumption one is that the effect of confounders on crimes shown on police.uk does not change over time $P(C_{g}|U,T)=P(C_{g}|U)$. Where $U$ are either the Easting or Northing coordinates of the house. Since we do not have SYP data yet, we cannot generate $C_{g}$ for the year 2010.

However, we can check assumption two and three: - $P(M_{s}|U,T)=P(M_{s}|U)$ - $P(U|T)=P(U)$

We can simply check this for the years 2010 and 2011 graphically:


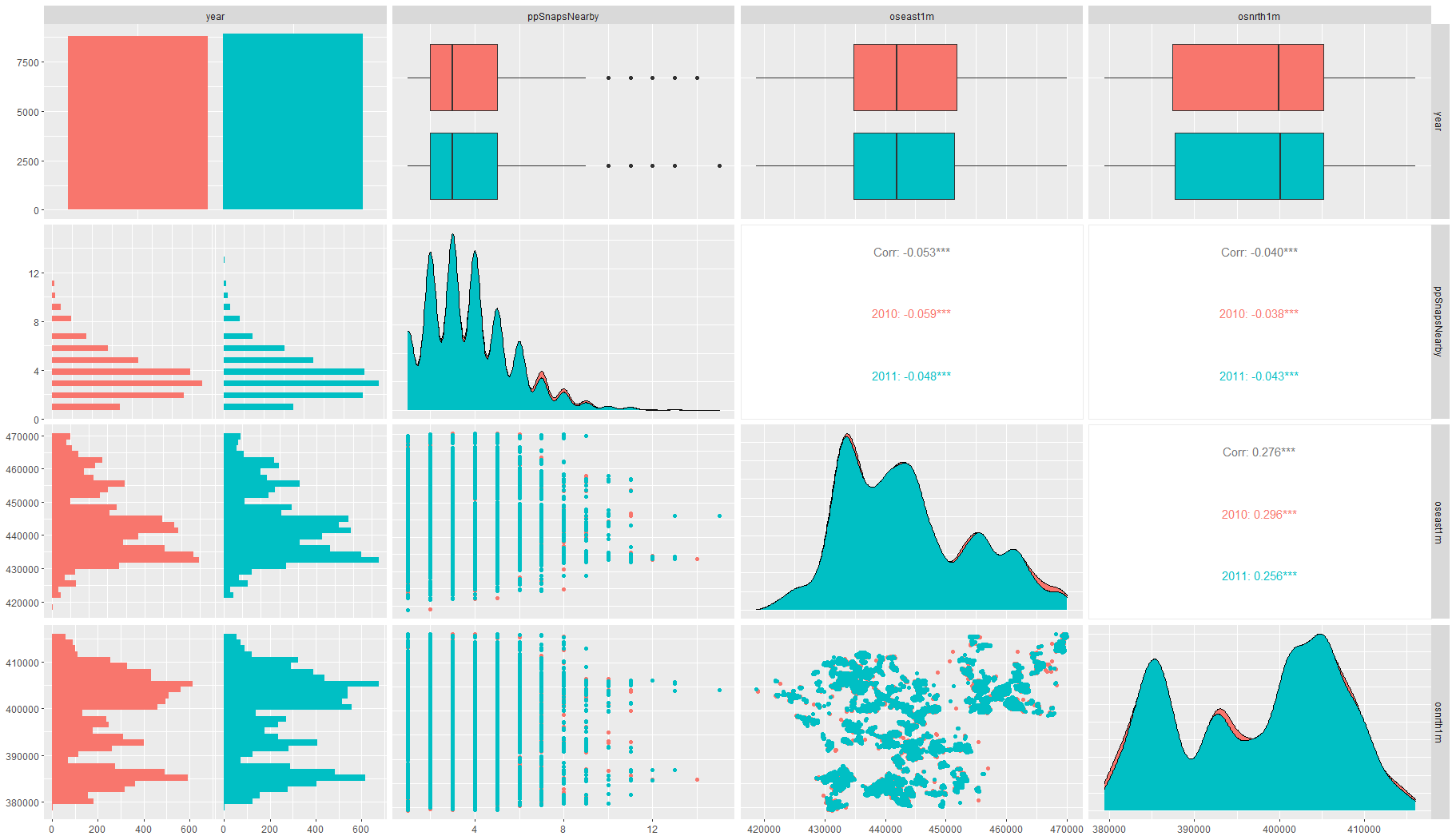
 **Fig S1.2: Pairwise summaries of variables**

Across the 2010 and 2011, the underlying univariate and multivariate distribution of coordinates are very close. The relationship between these coordiantes and snaps nearby is also the same between 2010 and 2011.

Assumptions four and five must be checked in other ways (i.e investigating other sources of evidence).

For pre-intervention checks, we can show that the correlation between the sum of snaps nearby $M_{s}$ and (log) house prices do not change between 2009 and 2010. For estimates 1A and 1C, we have shown that the rank correlation and time trend remains stable between 2009 and 2010. For 1B, we can show that the interaction in the OLS estimate is not significant (see below):

t test of coefficients:

 Estimate Std. Error t value Pr(>|t|)
(Intercept) 11.8748855 0.0105098 1129.8865 < 2e-16 ***
ppSnapsNearby -0.0501316 0.0023814 -21.0509 < 2e-16 ***
I(year == 2010)TRUE 0.0324777 0.0150241 2.1617 0.03066 *
ppSnapsNearby:I(year == 2010)TRUE -0.0039495 0.0034396 -1.1483 0.25088
---
Signif. codes: 0 ‘***’ 0.001 ‘**’ 0.01 ‘*’ 0.05 ‘.’ 0.1 ‘ ’ 1
