# Supplementary material for: Estimating the effects of crime maps on house prices using an (Un)natural experiment: A study protocol
Source: PLoS One. 2022 Dec 1;17(12):e0278463. doi: 10.1371/journal.pone.0278463 (PMC9714891; doi:10.1371/journal.pone.0278463)
Supplement: S2 File — (DOCX) [file pone.0278463.s002.docx]

## S2. DAG with more confounders

Additional variables are:

- $I$ represent entire information sets/ datasets with a subscript denoting different information.
  - $I_{s}$ is the secret list of snap-points used by police.uk. We know how $I_{s}$ was constructed (see supplement S3).
  - $I_{c}$ represents crimes, where they occur and crime type. The level of crimes around a house (summarised by $C_{r}$) affects house prices in various ways (e.g. property destruction).
  - $I_{h}$ represents houses and their characteristics, including their location.

Other variables retain the same meaning. The simpler DAG in the main text treats $I$ as items in the larger set of confounders $U$.


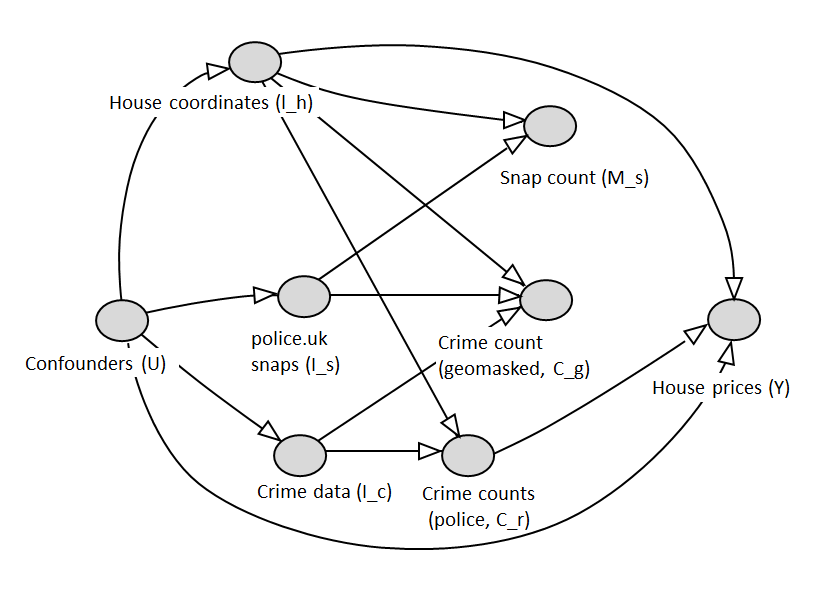


**Fig S2.1: DAG/ Path diagram of causal relations during the control period**

The purpose of this DAG is to show that:

1. $M_{s}$ is purely a function of only two inputs: $I_{h}$ and $I_{s}$.
2. Crime count shown $C_{g}$ on police.uk is a function of only three inputs: $I_{h}$, $I_{s}$, and $I_{c}$
3. The pathway between map features $M_{s}$ and $C_{g}$, and all other confounders $U$ are intercepted by $I_{h}$, $I_{s}$, and $I_{c}$. Therefore three information sets are the key sources of confounding.
