# Supplementary material for: Estimating the effects of crime maps on house prices using an (Un)natural experiment: A study protocol
Source: PLoS One. 2022 Dec 1;17(12):e0278463. doi: 10.1371/journal.pone.0278463 (PMC9714891; doi:10.1371/journal.pone.0278463)
Supplement: S3 File — (DOCX) [file pone.0278463.s003.docx]

# S3. Materials related to police.uk

## Geomasking in police.uk

The geomasking routine currently used on police.uk is documented on data.police.uk. Using information from various sources and public data, we know that the geomasking routine was changed in 2012 when police.uk updated its snap-points list. To summarise, the data processing for police.uk is as follows:

1. Home Office receives geocoded data from individual police forces and performs data checks (e.g. identifying crime outside police force boundaries).
2. The coordinates of crimes and incidents are changed to the coordinates of the nearest snap-point for the public data.

We note that police.uk crime maps were updated with a 2-month delay until at least 2016 [1]. The list of potential snap points used in step two is a secret. Between 2011 to 2013, we believe there were at least two major versions of the snap-point list which we henceforth refer to as Version One and Version Two. Version One was used on records between December 2010 and November 2011 whilst Version Two was used on records from December 2011 until at least the end of our study period (December 2013). The snap points were created in the following ways:

*Version One*: This was created in 2011 using Ordnance Survey data (exact datasets unverified). Potential snap-points were the centre of residential roads (see p. 93 - 94 in [2]), and Catchment areas for each point were created using Voronoi polygons (Fig 3.1). If a snap-point has less than 12 postal addresses within its catchment, it is dropped. This version consists of the remaining snap-points. For computational efficiency, police.uk could have used a nearest neighbour finding algorithm to do the postal address counting. This would have yielded the same outcome as the catchment approach.


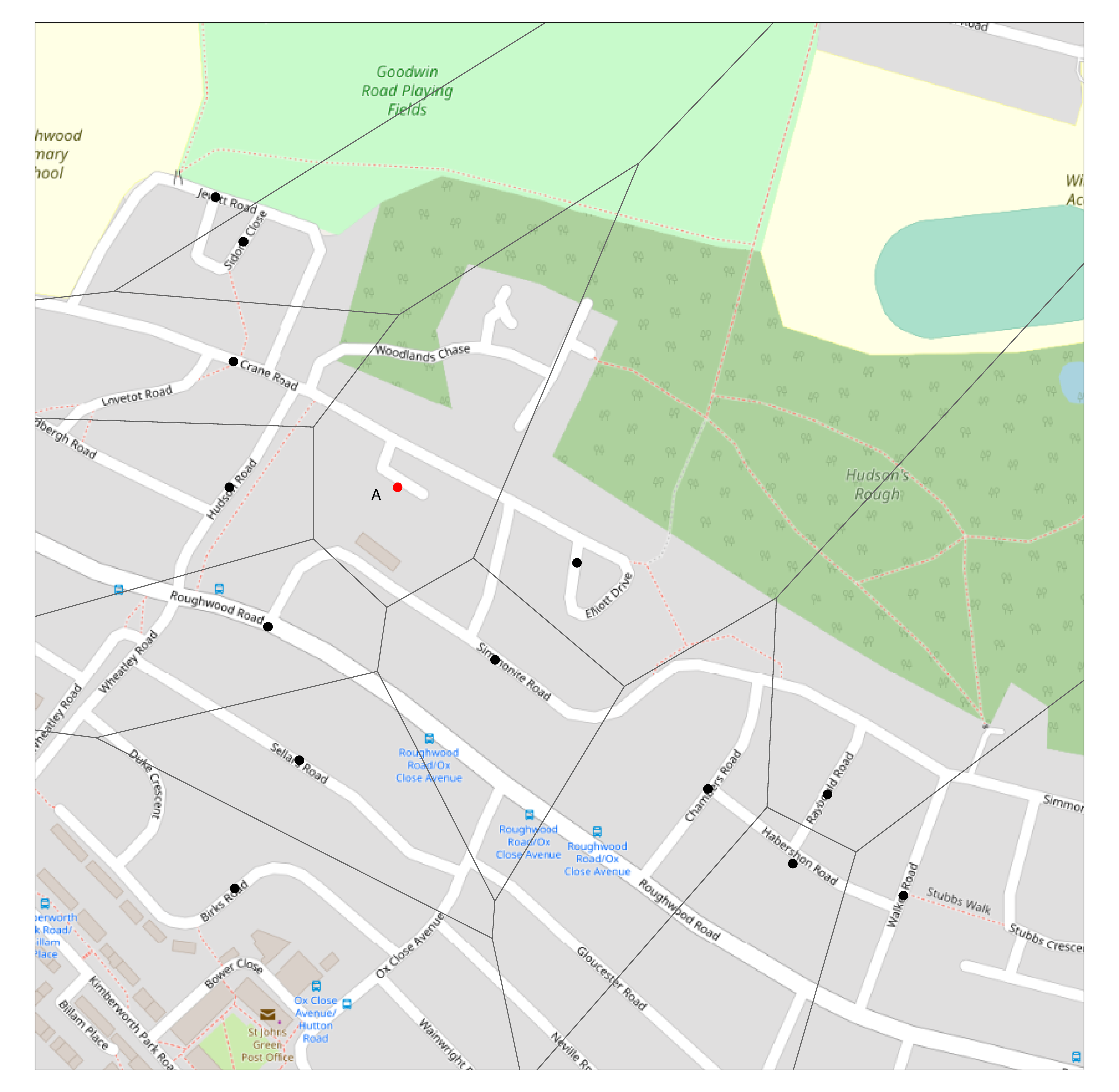


**Fig S3.1. Example of Voronoi polygons.** Polygon A is associated with point A and represents areas closer to point A than any other point. Source: OpenStreetMap (base map only).

*Version Two*: These snap-point list were created in 2012. The centre point of every road in England and Wales was taken from the Ordnance Survey Locator dataset. These points were augmented with locally relevant points of interest from the Point X dataset. Points of interest include public places, such as parks, or commercial premises, such as shopping centres. Each map point was analysed to see how many postal addresses were contained in its catchment area according to the Ordnance Survey Address-Point dataset. Again, the catchments were created using Voronoi polygons. Any catchments with between 1 and 7 postal addresses were discarded to protect privacy. The remaining points were provided to police forces for human assessment; a small number of additions and deletions were made to make map points more locally relevant.

Our main source of information regarding these snap points are police.uk itself and its accompanying data website: data.police.uk [3] [4]. Information on Version One was inferred from [2] and cross-referenced with information from older versions of data.police.uk. We inferred the timing of the version from looking at the dates that unique points were first recorded on police.uk. For both versions, the locations of snap-points are based on the location of static urban features. The centre of streets form the vast majority of snap-points in Version Two and the entirety of Version One.

In theory, since police.uk used Ordnance Survey data, we can recreate the majority of snap-points in their master snaps list. In practice, given the density of crime in urban regions and a long enough timeframe, all the unique snap-points on police.uk will eventually be used. So we can infer the master snaps list by counting the number of unique locations used by police.uk during a given period. Using this method, we can recover 96% of all the snap-points used in the 2012 and the vast majority used in Version One.

# Inferring police.uk’s list of snaps

As mentioned earlier, there are two major versions of the snap list. Version one was in use until 2012, and version two was used afterwards. The creation of both versions uses roughly the same routine:

1. Get a list of urban features (mostly centre of residential roads) from Ordinance survey data. Add in additional locations suggested by the local police forces. These form the initial list of snap-points.
2. For each snap-point, construct catchments using Voronoi polygons. Apply a privacy filter by omitting snap points with catchments containing fewer than a certain number of addresses. The remaining snap-points form the final snap-point list.

For version two, we know there were 762,000 potential snaps in use in 2013 by police.uk (see table below from data.police.uk). Before applying the privacy filter in step 2, there were 955,000 potential snap-points. So we know that a substantial proportion of changes were due to the privacy filtering routine.

|  | final count | count before privacy filters |
| --- | --- | --- |
| Street | 679089 | 833913 |
| Sports/Recreation Area | 24510 | 34237 |
| Parking Area | 17797 | 29591 |
| Park/Open Space | 14051 | 20418 |
| Supermarket | 5703 | 7518 |
| Petrol Station | 5501 | 7296 |
| Pedestrian Subway | 4570 | 6173 |
| Shopping Area | 3024 | 4232 |
| Further/Higher Educational Building | 1347 | 2095 |
| Police Station | 1083 | 1605 |
| Hospital | 982 | 1721 |
| Nightclub | 824 | 1109 |
| Bus/Coach Station | 816 | 1141 |
| Theatre/Concert Hall | 733 | 997 |
| Conference/Exhibition Centre | 524 | 781 |
| Airport/Airfield | 369 | 564 |
| Added by Police Force | 350 | 577 |
| Ferry Terminal | 203 | 320 |
| Theme/Adventure Park | 106 | 370 |
| Prison | 102 | 171 |
| Race Track | 100 | 170 |
| Motorway Service Area | 79 | 147 |
| sum | 761863 | 955146 |
| Source: <https://data.police.uk/about/> (2013) |  |  |

**Table S3.1: Total snap-points used by police.uk (source: data.police.uk)**

As mentioned before, given the density of crime in urban regions and a long enough period, all the unique snap points on police.uk will eventually be used. So we can simply infer the master snap list by counting the number of unique locations used by police.uk during a given period. To check for changes to the master snaps, we studied the dates when each snap was first used. We found a large influx of new snaps being used for the December 2011 data (see Fig S3.2 below).


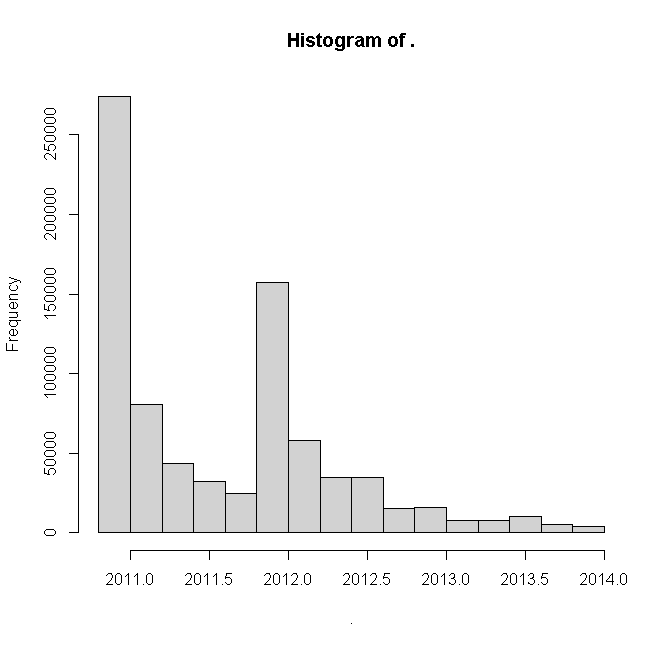


**Figure S3.2. Histogram of data of the first appearance of each snap point**

This and other evidence [2] allow us to pinpoint when version two first came into use (i.e. for police data from December 2011).

# References

1. Quinn A, Cooke L, Monaghan M. An exploration of the progress of open crime data: How do ongoing limitations with the Police.Uk website restrict a comprehensive understanding of recorded crime? Policing and Society. 2019;29: 455–470. doi:[10.1080/10439463.2017.1397149](https://doi.org/10.1080/10439463.2017.1397149)

2. Smith AM, Heath, Tom. Police.Uk and Data.Police.Uk: Developing Open Crime and Justice Data for the UK. JeDEM - eJournal of eDemocracy and Open Government. 2014;6: 87–96. doi:[10.29379/jedem.v6i1.326](https://doi.org/10.29379/jedem.v6i1.326)

3. About | data.police.uk. https://data.police.uk/about/;

4. About | data.police.uk. https://web.archive.org/web/20130705204237/https://data.police.uk/about/; Jul 2013.
